# Temporal trends in educational inequalities in non-communicable diseases in Korea, 2007-2015

**DOI:** 10.1371/journal.pone.0190143

**Published:** 2017-12-28

**Authors:** Gyu Ri Kim, Chung Mo Nam

**Affiliations:** 1 Department of Biostatistics, Graduate School of Public Health, Yonsei University, Seoul, Korea; 2 Department of Preventive Medicine, College of Medicine, Yonsei University, Seoul, Korea; CUNY, UNITED STATES

## Abstract

**Background:**

Socioeconomic inequalities in non-communicable diseases are known to exist; however there is a paucity of research describing the secular trends in these inequalities. To this end, the current study aims to explore the recent time trends in social patterning of selected non-communicable diseases among Korean adults between 2007 and 2015.

**Methods:**

Using data from the Korea National Health and Nutrition Examination Survey (KNHANES), temporal trends in socioeconomic inequalities in diabetes, arthritis, asthma and depressive symptoms were assessed across three time points. Respondents were adults aged 20 years or over (N = 47,091, 20,180 men and 26,911 women). Socioeconomic circumstance was assessed based on highest level of educational attainment. We estimated prevalence ratios with 95% confidence intervals using Poisson regression with robust variance estimation (adjusted for age, smoking status, alcohol consumption, obesity, and physical activity) separately for men and women. The magnitude of the inequalities was computed using the relative index of inequality (RII).

**Results:**

In men, diabetes was not associated with educational attainment, while there was evidence of a negative association in women across surveys. Similar inverse associations were found with arthritis and depressive symptoms, but these associations were less clear for asthma. RII showed a non-significant increasing trend in educational disparities in depressive symptoms. Meanwhile, relative inequalities in diabetes, arthritis and asthma have narrowed. These trends were, in general, more pronounced in women.

**Conclusions:**

The findings of this study indicate higher burden of selected NCDs among the lower educational groups, particularly among women. In addition, our results indicated some improvements in inequalities in diabetes, arthritis and asthma in recent years. These findings have important implications for understanding the causes of social patterning of NCDs and for the targeting of effective interventions.

## Introduction

Cardiovascular disease, diabetes, chronic respiratory diseases, and other non-communicable diseases (NCDs) are major public health concern, being the leading causes of mortality worldwide. Of the 56.4 million deaths that occurred globally in 2015, an estimated 39.5 million–approximately 70%–were due to NCDs and, by 2030, total deaths attributable to NCDs are projected to increase substantially to 52 million [1]. NCDs have long been considered as problem of high income countries; however, the percentage of total disease burden attributable to non-communicable is growing rapidly in the Asia-Pacific region [2]. In the case of Korea, NCDs are the leading contributors to mortality and disability-adjusted life years, accounting for almost 80% of all deaths and 85% of total disability-adjusted life-years (DALYs) lost in 2012 [3, 4].

Over the past few decades, Korea has experienced unprecedented economic development which has resulted in substantial unequal distribution of income opportunities, health care resources and deepening social polarization [5, 6]. In this context, tackling inequalities in health has featured prominently as a public health priority in recent years [7]. Much research has been devoted to elucidating the relationship between socioeconomic status and non-communicable diseases [8–12]. To date however, few studies have empirically examined the time trends in socioeconomic patterning of NCDs. In particular, existing studies have mostly focused on outcomes such as mortality [13–15], self-rated health [16, 17] and fatal diseases like cancer [18, 19] and cardiovascular diseases including stroke [20]. Yet the extent of and the trends in socioeconomic inequality with respect to nonfatal diseases, such as arthritis and asthma, despite being one of the leading contributors to Quality-Adjusted Life-years (QALYs) lost in Korea, have received relatively little attention [21]. Given the increasing burden of chronic diseases, monitoring the dynamics of the relationship between socioeconomic status (SES) and non-communicable diseases over time is imperative for development of targeted interventions aimed at tackling non-communicable diseases and improving ones’ quality of life. Thus, this study aims to investigate the recent trends in educational inequalities in selected non-fatal NCDs among Korean adults. Further, the gender differences in these inequalities were explored.

## Materials and methods

### Data

This study was based on data from the Korea National Health and Nutrition Examination Survey (KNHANES). KNHANES is an ongoing cross-sectional survey of non-institutionalized adults resident in Korea, commissioned by the Korea Centers for Disease Control and Prevention (KCDC). The first three KNHANES surveys (1998, 2001, 2005) were initially implemented as a tri-annual survey. Beginning with the fourth KNHANES in 2007, it was converted to an annual survey to provide timely national statistics on health [22]. The details of the sampling procedure and KNHANES survey response rates have been provided elsewhere [23]. In brief, the complete survey, which consists of three components (health interview, health examination survey, and a nutrition survey), covers a comprehensive range of topics including demographic and socio-economic characteristics, diet and nutrition, healthcare utilization, and general health status. To obtain a representative sample of the Korean population, this survey used a multi-stage stratified probability sampling procedure based on the geographic area and administrative districts drawn from household registries. All individuals voluntarily agreed to participate and provided written informed consent prior to enrollment.

For consistency in the methodology of data collection among the study periods, only data from years 2007 to 2015 were used in our analyses. The overall data was divided into three 3-year periods (2007–2009; 2010–2012; 2013–2015) to obtain robust estimates for each time period. From an original sample of 73,353 individuals, we excluded from our analysis individuals younger than 20 years (n = 17,969). An additional 8,293 cases were excluded due to missing data on outcomes and educational attainment. After these exclusions, total sample of 47,091 subjects (20,180 men; 26,911 women) were available for analysis. Compared with the analytic sample, those excluded from the sample because of missing data tended to be older (mean 53.5 years vs. 49.9 vs.; p<0.001), more likely to be male (45.1% vs. 42.9%; p<0.001), and have lower levels of formal schooling (Elementary education 43.8% vs. 23.2%;p<0.001).

#### Ethical statement

Data from the KNHANES survey are made publicly available through the KNHANES website (http://knhanes.cdc.go.kr). Thus, ethical approval was not required for this study.

### Variables

#### Definition of non-communicable diseases (NCD)

The main outcomes of interest in the study were diabetes mellitus, asthma, arthritis, and depressive symptoms. Presence of asthma and arthritis were ascertained through self-report of physician diagnosis of each disease during the health interview. Depressive symptoms were assessed through responses to a single-item question: “During the past 12 months, have you ever felt sad or hopeless and been unable to complete daily activities for two weeks or more?” (Yes or no). Diabetes was defined according to the following criteria: fasting plasma glucose levels ≥ 126 mg/dL, current use of anti-diabetic medications, or a previous diagnosis of diabetes by a physician.

#### Measure of socioeconomic circumstance

We used educational attainment as a measure of socioeconomic circumstances. In the KNHANES, the participants were asked the level at which their education was completed; for this analysis, participants were placed into four educational groups: (1) completion of elementary school, (2) middle school, (3) high school and (4) university level or above.

#### Covariates

Detailed information on the socio-demographic characteristics of participants was collected by trained staff through standardised questionnaires. Age was categorized as (1) 20–29 years, (2) 30–39 years, (3) 40–49 years, (4) 50–59 years, and (5) 60+ years. Among health-related behaviours, smoking, alcohol intake, physical activity, and obesity are typically more prevalent among those in the lower socioeconomic stratum and are associated with health-related outcomes [24–26]. We therefore considered them as covariates in our analysis. Participants were classified into three groups according to their smoking status: (1) Never smoker (Those who had never smoked or had smoked less than 100 cigarettes in their lifetime), (2) Former smoker (Those who reported smoking at least 100 cigarettes in their lifetime and who did not smoke at all at the time of the survey), (3) Current smokers (Subjects who reported smoking at least 100 cigarettes in their lifetime and who smoked either every day or on some days at the time of the survey). Participants were asked to indicate if they had consumed at least one glass of alcohol in their lifetime, recorded using a dichotomous (yes or no) scale. Physical activity was defined as engaging in moderate physical activity at least 5 days per week for more than 30 minutes each day or strenuous physical activity performed for at least 20 min at a time at least three times per week. Body mass index (BMI) was calculated as weight in kilograms divided by height in meters squared, and categorized in three groups using the criteria for Asians developed by the Western Pacific Region Office of the World Health Organization [27]. Specifically, individuals were classified as less than 18.5 kg/m^2^ (underweight), between 18.5 and 24.9 kg/m^2^ (normal weight), and more than 25 kg/m^2^ (obese).

### Statistical analysis

Previous studies have demonstrated significant gender differences in the association between socioeconomic status and chronic diseases [28–30]. Hence all analyses were performed separately for men and women. Distributions of study variables are summarized as weighted percentages for categorical variables and weighted means (standard errors) for continuous variables. Age-standardized estimates of NCD prevalence was initially computed for each time period, overall and according to socioeconomic groups, using the direct standardisation method. The Korean 2010 census population was used as the standard population. Subsequently, Poisson regression with robust variance estimation (a generalized linear model with a logarithmic link function and Poisson distribution) were used to estimate prevalence ratios (PRs) and 95% confidence intervals for each NCD across educational groups [31, 32]. By assuming that the observations are independent and log transformed outcomes are linearly related to the independent variable, exponentiation of the model coefficients yields the PR. PRs are preferred to odds ratios because they are more easily interpretable when evaluating exposure effect [33] and since it is known that odds ratios overestimate the PRs, particularly when the outcome is not rare [34]. Model 1 was adjusted for age only; in model 2, we additionally adjusted for potentially mediating behavioural risk factors (self-reported smoking status, alcohol consumption, obesity, and physical activity). The highest educational level group was used as the reference and P values of less than 0.05 were considered statistically significant. For the behavioural risk factors, there were small amounts of missing values, usually less than 1%. These observations were treated as a separate missing category and included in the models to enable inclusion of all participants.

In addition, the extent of educational inequalities in NCDs was measured using the relative index of inequality (RII) [35]. The RII is a continuous measure of educational inequalities that accounts simultaneously for the changing size and relative position of educational groups across time. Educational attainment is hierarchically ranked from highest to lowest and a ridit-score is derived for each stratum of educational level by calculating the mean proportion of the population with a higher SES. In our case, for instance, if the university level and high school education groups comprise 20% and 10% of the population, using midpoints for the proportion of the population in each stratum, the ridit-score for those with university level education would be 0.1 (0.2/2) and 0.25 (0.2 +(0.1/2)) for those attaining high school education only. Each of the outcomes was then regressed on these ridit-scores, with the resulting regression coefficient representing the RII. RII values larger than 1 imply that the prevalence of NCDs is higher in the lower socioeconomic group. Additionally, in order to assess the changes in RII across surveys, an interaction term of ridit-score*survey was included in each model. Also, gender differences in inequality for each survey were assessed by inserting two-way interaction terms (Ridit-score*sex), using men as the reference group. All statistical analyses were performed using survey estimation command (svy) of STATA, version 13 (StataCorp, College Station, TX) to incorporate sample weights and account for clusters and strata of the complex sample design.

## Results

### Sample characteristics

Table 1 gives the socio-demographic characteristics of participants in each KNHANES survey. The total sample comprised of 47,091 respondents, including 20,180 men and 26,911 women. Over the study period, there was a steady rise in the mean age for both sexes; mean age increased from 43.6 years (SD = 0.27) in 2007–2009 to 45.1 years (SD = 0.26) in 2013–2015, and from 45.4 years (SD = 0.28) in 2007–2009 to 46.7 years (SD = 0.27) in 2013–2015 for men and women, respectively. The proportion of women was greater than that of men in all surveys, with females constituting approximately 57% of the total sample. The majority of the participants were married, and physically inactive. Approximately one-third (between 36.3–38.4% males; 26.6–28.4% females) of the sample was classified as obese. The proportion of current smokers in the surveyed population ranged between 41.0% and 47.3% in men, and 5.7% and 7.0% in women, whereas the percentage of current drinkers among males and females ranged around 95% of men and 82–85% of women. In both men and women, the percentage of participants with elementary school education declined between 2007–2009 and 2013–2015, whilst those reporting a university education increased over the study period. Regarding the four selected NCDs, the weighted proportion of adults reporting diabetes ranged from 8.5% to 10.6% in men, and in women from 6.9% to 7.8%. For depressive symptoms, the values ranged between 5.3% to 9.3% in men and 10.2% to 18.6% in women. Among males, the crude prevalence of physician-diagnosed arthritis ranged from 3.0% to 4.2% across surveys, and in females from 12.9% to 13.2%, meanwhile prevalence estimates of asthma among men and women ranged from 2.2% to 2.6% and 3.1% to 3.4%, respectively.

**Table 1:** Sociodemographic characteristics of men and women aged 20 years or over in Korean national health and nutrition examination survey (KNHANES) according to study period.

	Men (n = 20,180)			Women (n = 26,911)		
	2007–2009	2010–2012	2013–2015	2007–2009	2010–2012	2013–2015
**All participants (N**^**a**^**)**	7,010	7,160	6,010	9,400	9,545	7,966
**Age, mean (SD)**	43.6 (0.27)	44.4 (0.28)	45.1 (0.26)	45.4 (0.28)	46.2 (0.26)	46.7 (0.27)
**Age group (years)**						
20–29	887 (20.5)	745 (19.3)	719 (19.4)	1,141 (18.7)	1,068 (17.2)	893 (16.4)
30–39	1,412 (23.4)	1,308 (21.9)	978 (20.1)	1,967 (21.8)	1,871 (20.8)	1,378 (19.4)
40–49	1,445 (23.4)	1,353 (23.0)	1,112 (21.5)	1,874 (22.5)	1,717 (21.8)	1,535 (21.7)
50–59	1,213 (16.7)	1,381 (18.7)	1,195 (20.3)	1,597 (16.2)	1,930 (18.9)	1,685 (20.5)
60+	2,053 (16.0)	2,373 (17.1)	2,006 (18.7)	2,821 (20.8)	2,959 (21.3)	2,475 (22.0)
**Education**						
Elementary	1,265 (11.7)	1,049 (9.9)	788 (8.7)	3,139 (25.3)	2,740 (22.7)	1,920 (18.4)
Middle school	905 (10.4)	856 (9.6)	682 (8.4)	1,056 (10.9)	1,091 (10.9)	910 (9.9)
High school	2,061 (30.6)	2,144 (31.4)	1,774 (29.4)	2,707 (32.4)	2,623 (30.4)	2,263 (30.2)
University level or above	2,779 (47.3)	3,111 (49.1)	2,766 (53.5)	2,498 (31.4)	3,091 (36.0)	2,873 (41.5)
**Diabetes**						
Yes	725 (8.5)	874 (9.1)	820 (10.6)	762 (6.9)	789 (7.0)	757 (7.8)
**Arthritis**						
Yes	398 (4.2)	328 (3.0)	323 (3.7)	1,576 (13.2)	1,584 (13.0)	1,328 (12.9)
**Asthma**						
Yes	184 (2.2)	207 (2.6)	139 (2.3)	340 (3.1)	317 (3.1)	286 (3.4)
**Depressive symptoms**						
Yes	737 (9.3)	632 (9.0)	343 (5.3)	1,847 (18.6)	1,550 (16.4)	819 (10.2)
**Marital status**						
Never married	1,119 (23.3)	704 (17.6)	1,079 (26.7)	976 (14.6)	730 (11.4)	991 (16.8)
Married/cohabiting	5,453 (71.0)	5,759 (70.0)	4,542 (68.1)	6,435 (67.6)	6,785 (68.4)	5,543 (68.7)
Divorced separated/ widowed	402 (4.9)	324 (4.2)	380 (5.1)	1,963 (17.4)	1,640 (14.8)	1,425 (14.4)
Missing	36 (0.8)	373 (8.2)	9 (0.1)	26 (0.4)	390 (5.4)	7 (0.1)
**Smoking status**						
Never smoker	1,278 (19.0)	1,287 (19.5)	1,304 (24.0)	8,273 (86.5)	8,532 (86.6)	7,131 (88.3)
Former smoker	2,638 (33.7)	2,920 (33.8)	2,404 (34.7)	553 (6.7)	504 (6.4)	409 (5.7)
Current smoker	3,093 (47.3)	2,951 (46.7	2,281 (41.0)	573 (6.8)	509 (7.0)	398 (5.7)
Missing	1 (0.0)	2 (0.0)	21 (0.3)	1 (0.0)	0 (0.0)	28 (0.3)
**Alcohol consumption**						
No	399 (4.8)	350 (4.1)	290 (4.0)	2,011 (18.4)	1,893 (15.9)	1,374 (14.6)
Yes	6,611 (95.2)	6,809 (95.9)	5,700 (95.7)	7,387 (81.5)	7,652 (84.1)	6,572 (85.2)
Missing	0 (0.0)	1 (0.0)	20 (0.3)	2 (0.0)	0 (0.0)	20 (0.2)
**Physical activity**						
No	5,048 (73.2)	5,627 (77.0)	5,535 (91.6)	7,205 (77.7)	7,956 (83.3)	7,552 (94.6)
Yes	1,962 (26.8)	1,533 (23.0)	475 (8.4)	2,195 (22.3)	1,589 (16.7)	414 (5.4)
Missing	0 (0.0)	0 (0.0)	0 (0.0)	0 (0.0)	0 (0.0)	0 (0.0)
**Obesity**						
Underweight (< 18.5 kg/m^2^)	247 (3.3)	183 (2.4)	139 (2.5)	485 (6.2)	528 (6.6)	407 (6.3)
Normal (18.5 to 24.9 kg/m^2)^	4,278 (59.9)	4,418 (60.7)	3,568 (59.0)	6,098 (65.7)	6,195 (64.9)	5,258 (67.0)
Obese (>25 kg/m^2)^	2,449 (36.3)	2,538 (36.6)	2,296 (38.4)	2,777 (27.6)	2,810 (28.4)	2,296 (26.6)
Missing	36 (0.5)	21 (0.3)	7 (0.1)	40 (0.5)	12 (0.1)	5 (0.1)

^a^Unless otherwise stated, results are expressed as unweighted frequencies with weighted percentages shown in brackets.

### Age-standardised estimates of NCDs prevalence

**Fig 1:**
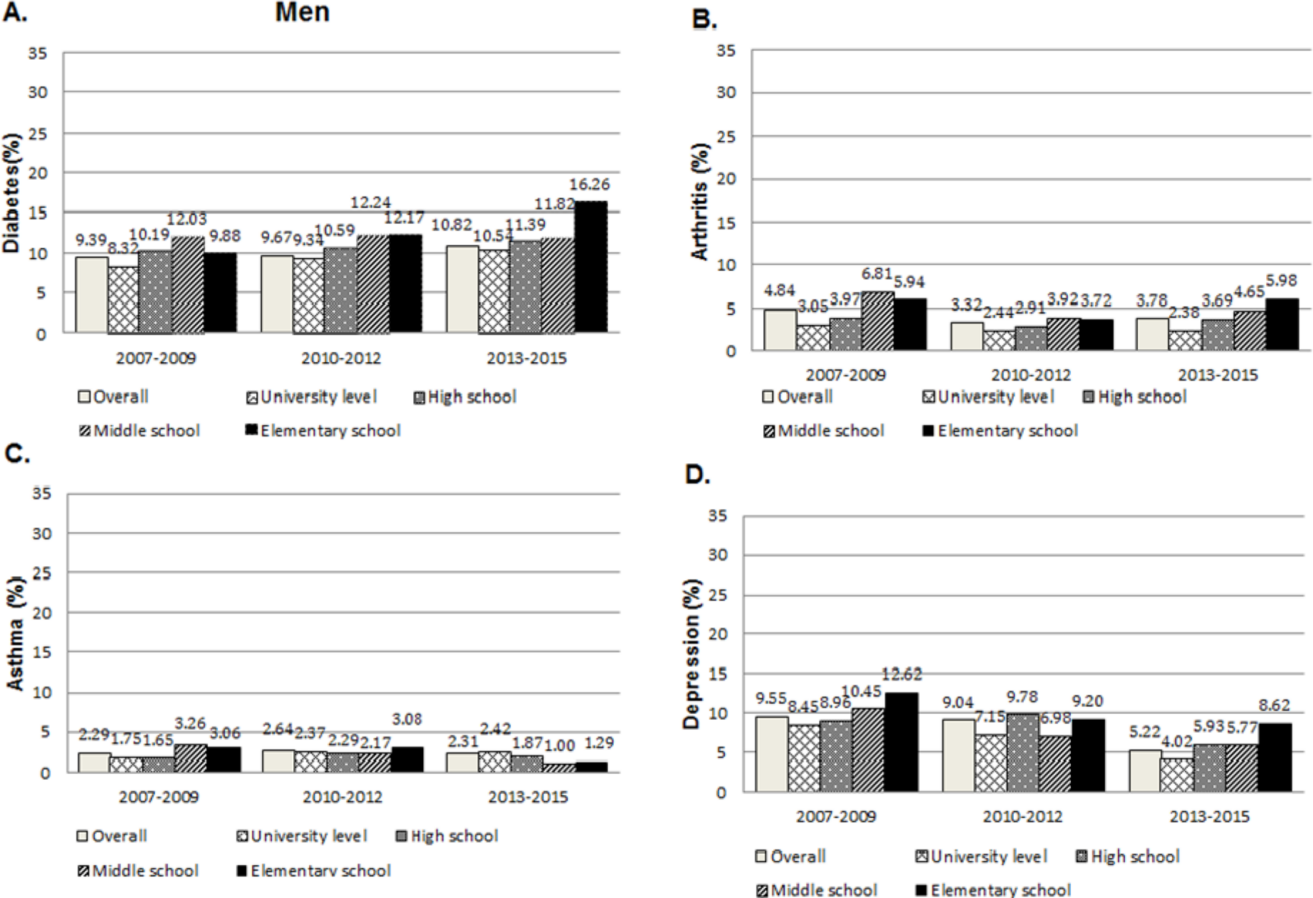
Age-standardised prevalence and 95% CI of NCDs in men aged 20 or over, according to educational level–KNHANES between 2007–2015.

**Fig 2:**
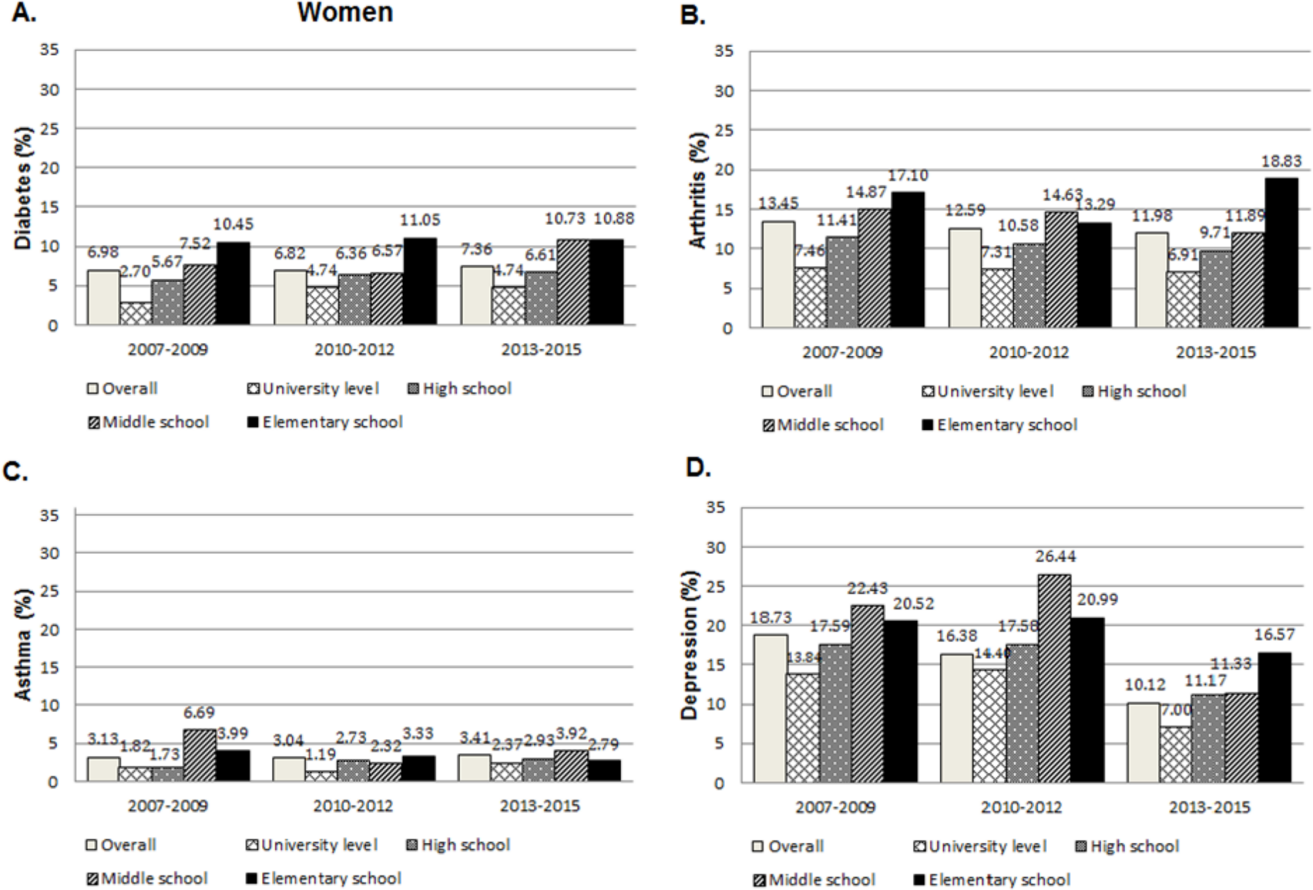
Age-standardised prevalence and 95% CI of NCDs in women aged 20 or over, according to educational level–KNHANES between 2007–2015.

Figs 1 and 2 illustrate the age-standardised prevalence estimates (%) with 95% confidence intervals for each non-communicable disease by educational level. In men, the overall age-standardised prevalence of diabetes increased from 9.39 (95% CI 8.56 to 10.23) in 2007–2009 to 10.82% (95% CI 10.02 to 11.63) in 2013–2015 (Fig 1A). On the contrary, the overall prevalence of diabetes did not change significantly in women (Fig 2A). Prevalence of asthma remained relatively stable over the study period at around 2% and 3% for men and women, respectively. In general, there were downward trends with respect to arthritis and depressive symptoms, with overall prevalence decreasing in both men and women. For most NCDs, prevalence estimates generally declined with increasing educational level. The clearest pattern of relationship was seen among women and in 2013–2015. However, no consistent pattern was seen in the prevalence of asthma across the survey period.

Tables 2 and 3 present the prevalence ratios and their confidence intervals for the associations between level of educational attainment and NCDs. There has no significant association between diabetes and education among men. Women, on the other hand, showed a higher prevalence of diabetes in individuals with elementary education than their more educated counterparts, with age-adjusted PRs ranging from 1.99 to 4.90 (p for trend <0.001). Our data indicated a clear inverse gradient in prevalence of arthritis across educational groups for both genders, with higher prevalence amongst the subjects with lower educational attainment. These associations were significant for all survey years. For example, relative to those with university level education or above, the age-adjusted PRs of arthritis for the elementary education group in 2013–2015 was 2.74 (95% CI 2.05 to 3.66; p trend<0.001) among women and 2.60 (95% CI 1.70 to 3.98; p for trend <0.001) among men. Moreover, the results were generally robust to adjustments for smoking status, alcohol consumption, physical activity and obesity, although slight attenuations of the association were observed (Table 2, Model 2). The estimated prevalence ratios for asthma tended to be greater among the lowest education group at different time points, although some exceptions were noted. For example, in men, Poisson regression revealed non-significant association of education with asthma in 2013–2015.

**Table 2:** Prevalence ratios and 95% confidence interval for NCDs among men, by educational level and survey year from 2007 to 2015.

	2007–2009		2010–2012		2013- 2015	
	Model 1^†^	Model 2^‡^	Model 1^†^	Model 2^‡^	Model 1^†^	Model 2^‡^
**Diabetes**						
Elementary school	1.11 (0.81 to 1.53)	1.15 (0.84 to 1.60)	1.14 (0.86 to 1.50)	1.11 (0.84 to 1.47)	1.14 (0.89 to 1.45)	1.11 (0.87 to 1.42)
Middle school	1.38 (1.02 to 1.88)	1.40 (1.03 to 1.91)	1.45 (1.09 to 1.91)	1.39 (1.05 to 1.83)	1.19 (0.92 to 1.55)	1.16 (0.90 to 1.52)
High school	1.32 (1.04 to 1.66)	1.30 (1.03 to 1.65)	1.41 (1.13 to 1.75)	1.37 (1.09 to 171)	1.28 (1.05 to 1.55)	1.24 (1.02 to 1.50)
University level or above	Ref.	Ref.	Ref.	Ref.	Ref.	Ref.
p for trend	0.478	0.331	0.298	0.396	0.331	0.438
**Arthritis**						
Elementary school	2.70 (1.75 to 4.16)	2.77 (1.78 to 4.31)	1.52 (1.03 to 2.23)	1.57 (1.07 to 2.33)	2.60 (1.70 to 3.98)	2.62 (1.71 to 4.04)
Middle school	2.23 (1.47 to 3.43)	2.22 (1.47 to 3.48)	1.75 (1.14 to 2.69)	1.76 (1.15 to 2.71)	2.31 (1.42 to 3.76)	2.32 (1.41 to 3.78)
High school	1.52 (0.93 to 2.47)	1.51 (0.92 to 2.47)	1.15 (0.74 to 1.76)	1.17 (0.76 to 1.80)	1.85 (1.22 to 2.79)	1.85 (1.22 to 2.81)
University level or above	Ref.	Ref.	Ref.	Ref.	Ref.	Ref.
p for trend	<0.001	<0.001	**0.010**	0.006	<0.001	<0.001
**Asthma**						
Elementary school	2.14 (1.22 to 3.77)	2.22 (1.28 to 4.06)	2.47 (1.35 to 4.52)	2.43 (1.32 to 4.45)	1.17 (0.58 to 2.37)	1.21 (0.59 to 2.46)
Middle school	1.45 (0.88 to 2.38)	1.56 (0.92 to 2.58)	1.47 (0.75 to 2.88)	1.47 (0.75 to 2.89)	0.97 (0.53 to 1.76)	0.98 (0.54 to 1.80)
High school	0.78 (0.49 to 1.26)	0.82 (0.51 to 1.34)	0.81 (0.49 to 1.32)	0.81 (0.50 to 1.32)	0.68 (0.40 to 1.14)	0.69 (0.41 to 1.17)
University level or above	Ref.	Ref.	Ref.	Ref.	Ref.	Ref.
p for trend	0.007	0.004	**0.016**	0.018	0.947	0.958
**Depressive symptoms**						
Elementary school	1.79 (1.32 to 2.43)	1.74 (1.28 to 2.35)	1.87 (1.34 to 2.62)	1.75 (1.25 to 2.45)	2.78 (1.83 to 4.20)	2.75 (1.82 to 4.18)
Middle school	1.30 (0.96 to 1.77)	1.25 (0.93 to 1.70)	1.81 (1.32 to 2.46)	1.73 (1.27 to 2.37)	2.25 (1.43 to 3.56)	2.25 (1.44 to 3.56)
High school	1.12 (0.90 to 1.40)	1.08 (0.86 to 1.35)	1.36 (1.07 to 1.75)	1.30 (1.02 to 1.67)	1.58 (1.17 to 2.13)	1.56 (1.15 to 2.11)
University level or above	Ref.	Ref.	Ref.	Ref.	Ref.	Ref.
p for trend	<0.001	0.003	<0.001	<0.001	<0.001	<0.001

†Model 1: Adjusted for age.

‡Model 2: Adjusted for age, smoking status, alcohol consumption, physical activity and obesity.

**Table 3:** Prevalence ratios and 95% confidence interval for NCDs among women, by educational level and survey year from 2007 to 2015.

	2007–2009		2010–2012		2013- 2015	
	Model 1^†^	Model 2^‡^	Model 1^†^	Model 2^‡^	Model 1^†^	Model 2^‡^
**Diabetes**						
Elementary school	4.90 (2.94 to 8.19)	4.41 (2.69 to 7.23)	3.75 (2.29 to 6.13)	3.35 (2.07 to 5.42)	1.99 (1.39 to 2.86)	1.80 (1.26 to 2.58)
Middle school	3.88 (2.37 to 6.33)	3.56 (2.19 to 5.77)	3.48 (2.19 to 5.54)	3.19 (2.02 to 5.05)	2.11 (1.48 to 3.01)	1.93 (1.35 to 2.76)
High school	2.75 (1.76 to 4.29)	2.62 (1.68 to 4.07)	2.73 (1.79 to 4.16)	2.56 (1.68 to 3.89)	1.58 (1.16 to 2.14)	1.51 (1.10 to 2.05)
University level or above	Ref.	Ref.	Ref.	Ref.	Ref.	Ref.
p for trend	<0.001	<0.001	<0.001	<0.001	<0.001	0.003
**Arthritis**						
Elementary school	3.45 (2.38 to 4.99)	3.20 (2.24 to 4.57)	3.68 (2.59 to 5.24)	3.33 (2.35 to 4.70)	2.74 (2.05 to 3.66)	2.57 (1.93 to 3.41)
Middle school	3.64 (3.54 to 5.22)	3.39 (2.38 to 4.83)	4.11 (2.95 to 5.74)	3.74 (2.69 to 5.19)	2.60 (1.96 to 3.46)	2.41 (1.81 to 3.21)
High school	1.83 (1.31 to 2.54)	1.77 (1.27 to 2.46)	2.24 (1.65 to 3.05)	2.09 (1.54 to 2.85)	1.74 (1.33 to 2.26)	1.69 (1.30 to 2.20)
University level or above	Ref.	Ref.	Ref.	Ref.	Ref.	Ref.
p for trend	<0.001	<0.001	<0.001	<0.001	<0.001	<0.001
**Asthma**						
Elementary school	2.28 (1.26 to 4.14)	2.08 (1.15 to 3.74)	2.16 (1.26 to 3.69)	2.10 (1.22 to 3.62)	2.15 (1.47 to 3.16)	2.06 (1.39 to 3.04)
Middle school	1.46 (0.76 to 2.79)	1.36 (0.72 to 2.58)	1.55 (0.92 to 2.60)	1.51 (0.89 to 2.58)	1.34 (0.79 to 2.27)	1.30 (0.77 to 2.18)
High school	0.76 (0.48 to 1.20)	0.70 (0.44 to 1.12)	1.13 (0.72 to 1.76)	1.10 (0.70 to 1.73)	0.87 (0.58 to 1.31)	0.85 (0.57 to 1.27)
University level or above	Ref.	Ref.	Ref.	Ref.	Ref.	Ref.
p for trend	0.002	0.004	0.004	0.005	<0.001	<0.001
**Depressive symptoms**						
Elementary school	1.95 (1.55 to 2.45)	1.89 (1.50 to 2.37)	1.81 (1.44 to 2.27)	1.67 (1.34 to 2.08)	2.95 (2.19 to 3.98)	2.75 (2.04 to 3.70)
Middle school	1.63 (1.34 to 1.98)	1.61 (1.32 to 1.97)	1.63 (1.31 to 2.01)	1.51 (1.23 to 1.86)	1.89 (1.39 to 2.56)	1.82 (1.34 to 2.47)
High school	1.24 (1.04 to 1.48)	1.20 (1.01 to 1.42)	1.24 (1.06 to 1.46)	1.17 (0.99 to 1.38)	1.49 (1.20 to 1.87)	1.42 (1.14 to 1.77)
University level or above	Ref.	Ref.	Ref.	Ref.	Ref.	Ref.
p for trend	<0.001	<0.001	<0.001	<0.001	<0.001	<0.001

†Model 1: Adjusted for age.

‡Model 2: Adjusted for age, smoking status, alcohol consumption, physical activity and obesity.

### Relative index of inequality

Time trends in relative inequalities in NCDs are summarized in Table 4. Among women, the inequality in diabetes between educational levels appeared to decrease over time. The RII value decreased from 7.59 (4.04 to 14.22) in 2007–2009 to 2.58 (1.61 to 4.15) in 2013–2015 (Table 4), however, the test for overall trend in RII was not statistically significant (p = 0.096). Regarding gender differences, relative inequalities in diabetes were more marked among women than in men in all surveys (All P for gender interaction: p<0.001; see Table 5). For arthritis, the RII showed a consistent tendency towards reduced inequalities from 2007–2009 to 2013–2015 among women (p = 0.033) but not in men (p = 0.227). Furthermore, there was a significant ridit-score by gender interaction in 2010–2012 (P for gender interaction = 0.035). In relation to asthma, narrowing of educational inequalities was noted for both men and women, with significant differences in RII observed among males and females in 2010–2012 and 2013–2015. For depressive symptoms, RII showed a non-significant trend towards widening educational disparities in depressive symptoms (p for trend 0.156 in men and 0.881 in women) and no significant gender differences in RII were noted (all P values >0.05).

**Table 4:** Trends in educational inequalities in NCDs between 2007 and 2015.

**Men**	**2007–2009**	**2010–2012**	**2013–2015**	**P for trend across surveys†**
	**RII (95% CI)**	**RII (95% CI)**	**RII (95% CI)**	
Diabetes	1.37 (0.90 to 2.08)	1.46 (1.02 to 2.11)*	1.30 (0.94 to 1.80)	0.758
Arthritis	4.09 (2.21 to 7.62)***	2.01 (1.14 to 3.56)**	3.89 (2.15 to 7.07)***	0.227
Asthma	2.00 (0.94 to 4.26)	1.74 (0.76 to 4.00)	0.75 (0.33 to 1.69)	0.001
Depressive symptoms	1.81 (1.21 to 2.72)***	2.42 (1.57 to 3.75)***	3.82 (2.17 to 6.73)***	0.156
**Women**				
Diabetes	7.59 (4.04 to 14.22)***	5.10 (2.84 to 9.18)***	2.58 (1.61 to 4.15)***	0.096
Arthritis	5.79 (3.52 to 9.54)***	5.46 (3.57 to 8.36)***	3.98 (2.74 to 5.79)***	0.033
Asthma	3.17 (1.24 to 8.09)**	2.82 (1.30 to 6.09)**	2.51 (1.43 to 4.41)**	0.047
Depressive symptoms	2.50 (1.83 to 3.43)***	2.29 (1.67 to 3.14)***	4.24 (2.77 to 6.49)***	0.881

RII values larger than 1 indicate that the prevalence of NCDs is higher in the less educated group.

†P-values for the interactions between survey period and ridit-score.

* p<0.05.

**p<0.01.

*** p<0.001.

**Table 5:** Gender differences in relative index of inequality in NCDs.

	2007–2009	2010–2012	2013–2015
Diabetes	1.83 (1.31 to 2.34)	1.36 (0.92 to 1.80)	1.06 (0.69 to 1.43)
	<0.001*	<0.001*	<0.001*
Arthritis	1.73 (1.26 to 2.19)	1.60 (1.20 to 2.01)	1.37 (1.01 to 1.72)
	p = 0.416*	p = 0.035*	p = 0.925*
Asthma	1.12 (0.36 to 1.88)	1.54 (0.81 to 2.27)	1.17 (0.58 to 1.77)
	p = 0.437*	p = 0.003*	p<0.001*
Depressive symptoms	0.85 (0.57 to 1.13)	0.77 (0.49 to 1.05)	1.38 (0.98 to 1.79)
	p = 0.343*	p = 0.406*	p = 0.913*

* Results from the two-way interaction term—Ridit score* gender.

## Discussion

Using a representative sample of Korean adult population obtained by repeated cross-sectional surveys, we examined the trends in social distribution of diabetes, arthritis, asthma and depressive symptoms, among Korean adults between 2007 and 2015. A higher level of education was generally associated with more favorable outcomes; an exception was diabetes among men where no clear association with educational attainment was observed. This is in agreement with results reported by Imkampe and Gulliford who in the analyses of four repeated cross-sectional surveys in England between 1994 and 2006 found no significant relationship between educational level and diabetes men, but increasing relative inequalities women [36]. As has been shown by our study, numerous studies has documented inverse socioeconomic gradients in the incidence and prevalence of diabetes [37–40], arthritis [41], asthma [42, 43], and depression [44–46]. One common explanation given for the observed inverse educational gradient in NCDs is the variation in established risk factors of NCDs such as hypertension [47], obesity [48], smoking [49] and poorer dietary intake [50, 51], by educational level. Limited access to high-quality healthcare services among the less advantaged groups may also contribute to the inequalities in NCDs. In our data, however, the effect of education on NCDs remained significant after adjustment for age and multiple potentially mediating behavioral risk factors, suggesting that education as a risk factor for NCDs is at least partially independent of these covariates. In terms of secular trends, we observed trends of widening disparities in depressive symptoms over time, while inequalities have narrowed for diabetes, arthritis and asthma. Although there are no strictly comparable Korean studies on trends in educational inequalities in NCDs, our results are in accordance with previous research conducted by Hong et al. that demonstrated a widening pro-rich inequality in depression, which doubled over a 10 year period from 1997 to 2007 [52].

Another notable finding of the study is the significant gender differences in social patterning of NCDs. This corresponds with previous work demonstrating a steeper education gradient among women relative to men [53, 54]. The exact reasons for the observed gender heterogeneity remain uncertain. However, differences in exposure and vulnerability to material and behavioural risk factors have been postulated as possible explanations for the gender differences in social pattering of diabetes [55]. For example, it is well known that obesity, an important determinant of diabetes, is particularly prevalent among women with lower socioeconomic circumstances. Another likely explanation involves differentiated social roles, variations in exposure to stressors and coping strategies between men and women [56, 57]. Research has indicated that psychosocial stressors play an important role in determining health, but their effects are generally stronger for women relative to men. Also, women are typically are more involved in familial nurturing roles, while men’s roles are more instrumental. Thus, women are more likely to exposed to chronic stressors, which may in turn, be related to increased risk of developing NCDs. These explanations however remain speculative and merits further research.

There are a number of methodological limitations that limit the generalizability of our findings. First, the use of self-reported measures may have introduced recall error. For instance, the assessment of NCDs were based on self-reports, potentially leading to misclassification of disease status. Nonetheless, self-reported measures are frequently used in many population surveys for the surveillance of chronic diseases. Second, the sample sizes of the KNHANES were relatively small, especially after stratification by gender. Given the wide confidence intervals for the estimated RIIs, one must be cautious in generalizing the results. Further studies would benefit from a larger sample size to obtain more precise estimates of the trends in inequalities in NCDs and confirm the generalizability of our findings. A further limitation of this work is the possibility of selection bias due to the fall in response rates of KNHAES surveys in recent years [23]. Also, there is a potential bias through the decision to exclude those with missing information on education from our study. Since the analyzed participants were more likely to belong to the higher educational groups and were slightly younger than those excluded, we may have underestimated the prevalence of NCDs and possibly biased our results towards an underestimation of the association between NCDs and education. However, our estimate of self-reported NCD prevalence are comparable with reports using other sources of data [58, 59]. For example, according to the Korean National Health Insurance Service database (NHIS), diabetes prevalence for adults 30 years or older in 2012 was reported to be 7.1% and 8.5% for men and women, respectively. Despite these limitations, the present study also has several strengths. First, the use of a nationally representative sample surveyed over a 9-year period provided a comprehensive picture of the temporal trends in educational inequalities in non-communicable diseases among Korean adults. Second, because education is usually completed by early adulthood prior to the onset of chronic diseases, using educational attainment as a proxy for socioeconomic status has the advantage of minimizing the likelihood of reverse causality. Further, all surveys have been conducted according to standardised measurement protocol, which facilitated comparisons of social distribution of NCDs between different time points. To the best of our knowledge, this is one of very few studies examining the social patterning of non-communicable diseases in Korea. There is a need for prospective population-based studies designed to investigate underlying causes of these inequalities. This would improve the understanding of the development of inequalities in non-communicable diseases and could be beneficial for designing effective interventions strategies for Korean adults.

## Conclusions

In summary, the results of this study demonstrated variations in the association of non-communicable diseases and SEP by gender with a tendency for an inverse social gradient among Korean adults. Whilst educational inequalities in depressive symptoms remained relatively stable, educational differences in diabetes, arthritis, and asthma have generally narrowed over 9 years since 2007. These findings highlight the importance of gender-based population level interventions aimed at preventing NCDs.
